# Myoinositol and D-Chiro Inositol in Improving Insulin Resistance in Obese Male Children: Preliminary Data

**DOI:** 10.1155/2016/8720342

**Published:** 2016-11-01

**Authors:** Mario Mancini, Alice Andreassi, Michela Salvioni, Fiore Pelliccione, Gianna Mantellassi, Giuseppe Banderali

**Affiliations:** Pediatrics and Adolescent Andrological Project, Department of Pediatrics, San Paolo Hospital, Via di Rudini 8, University of Milan, 20142 Milan, Italy

## Abstract

Myoinositol and D-chiro inositol, which are inositol isomers, have been shown to possess insulin-mimetic properties and to improve insulin resistance, especially in women with polycystic ovary syndrome. However, it has not been determined if this relationship exists also in children. Based on these previous findings, we hypothesized that inositol could be effective in improving insulin sensitivity in children with insulin resistance. To evaluate this hypothesis, we administered both inositol formulations before carrying out an oral glucose tolerance test (OGTT) in a group of obese insulin-resistant male children with high basal insulin levels and compared the values obtained with an OGTT previously conducted without inositol, in the same group, with unchanged BMI. Our results confirm that myoinositol and D-chiro inositol acutely reduce insulin increase after glucose intake mainly in children with high basal insulin level.

## 1. Introduction

Myoinositol (MI) and D-chiro inositol (DCI) are isomeric forms of inositol that were found to have insulin-like properties, acting as second messengers in the insulin intracellular pathway; both of these molecules are involved in the increasing insulin sensitivity of different tissues to improve metabolic and ovulatory functions [1–3].

Myoinositol is the predominant form that can be found in nature and food. It is produced by the human body from D-glucose, but it is present in all living cells as membrane phospholipids and phytic acid. In food it is contained especially in pulses (beans, grains, and nuts) and fruits (in particular citrus fruits).

DCI and MI have different physiological roles since the former is crucial for glycogen synthesis while the latter increases cellular glucose uptake [4]. Each tissue has its own MI/DCI ratio, which is maintained through the conversion of myoinositol to D-chiro inositol occurring in tissues expressing the specific epimerase. High DCI levels are present in glycogen storage tissues, such as fat, liver, and muscle, whereas very low levels of DCI are typical of tissues with high glucose utilization, such as the brain and heart [5, 6].

The inositol is mainly catabolized by the kidney, which seems to be an important regulator of plasma inositol concentrations; however urinary excretion represents only a small fraction.

Indeed, deficiency or abnormalities in inositol metabolism induce a defect in glucose uptake and have been linked to insulin resistance and long term microvascular complications of diabetes [7].

A depletion of intracellular myoinositol and an excessive urinary excretion, along with lower level of chiro inositol and lower excretion, have been frequently observed in type II diabetic patients and in other conditions associated with insulin resistance, such as polycystic ovary syndrome, gestational diabetes, and metabolic syndrome. In general, certain data suggest that chiro inositol deficiency or imbalance is related more directly to insulin resistance itself, rather than to type II diabetes [4].

The chiro inositol deficiency observed in urine has also been confirmed in muscle and in blood, indicating a general defect in the body. This finding has provided the basis for initial trials of the administration of D-chiro inositol in STZ diabetic rats, monkeys, and subsequently humans [2, 8].

Thus administration of myoinositol and D-chiro inositol might play a role in restoring better insulin sensitivity, as well as delaying the onset of diabetes complications.

In this preliminary study obese male children have undergone an OGTT without inositol and then in association with inositol, in order to find out if the acute administration of inositol has a positive effect on insulin sensitivity.

We hypothesized that there may be a direct relation between higher BMI and a greater increase of insulin after glucose administration and that the effect of inositol might be different in subjects with higher BMI. We also proposed the hypothesis that there might be a relation between fasting insulin level and degree of insulin increase after glucose intake.

## 2. Materials and Methods

Twenty-three consecutive obese patients, aged 11.5 ± 2.3 (range 7–15), with a mean BMI of 29.8 ± 3.1 kg/m^2^ (mean ± SD) were recruited. Obesity was normalized according to Cacciari graphic percentiles [9].

Exclusion criteria were delayed puberty, hypogonadism (such as Klinefelter syndrome), thyroid dysfunctions, and obesity-linked genetic disease.

Anthropometric measures, like waist-hip ratio and percent of body fat, blood pressure, and blood samples for determination of total cholesterol, LDL, HDL, and triglycerides, were assessed. Blood samples for glucose and insulin were collected before and after the OGTT. Baseline patient characteristics are shown in Table 1.

All patients consumed a normocaloric diet, balanced for macronutrient distribution, in accordance with the national guidelines for the treatment of childhood obesity [10, 11]. Specifically, it is recommended that children follow, for a 6-month period, a normocaloric diet (daily caloric intake by age and sex [12]) consisting of protein (12%–15%), carbohydrates (55%–60%), fat (25%–30%; <10% saturated fatty acids, polyunsaturated fatty acids up to 10%, and monounsaturated fatty acids up to 15%), and fiber (range: age (year) plus 5 g-age (year) plus 10) [11, 12].

Additionally, it was recommended that children engage in at least 60 min of moderate-to vigorous-intensity physical activity daily, based on walking and tailored to individual preference [13].

After nutritional intervention patients who recorded in the first OGTT fasting insulin ≥15 *μ*U/mL and thus are considered as insulin-resistant were submitted to a second OGTT, which was preceded by the administration of 1 soft gel capsule of inositol [myoinositol 1100 mg + D-Chiro inositol 27.6 mg + folic acid 400 *μ*g; Inofolic Combi®, Lo.Li Pharma S.r.l., 00156 Rome, Italy]. In these insulin-resistant patients, blood samples for glucose and insulin were collected before and after the OGTT and data from the first and second OGTT were compared.

## 3. Statistical Analysis

**Figure 1:**
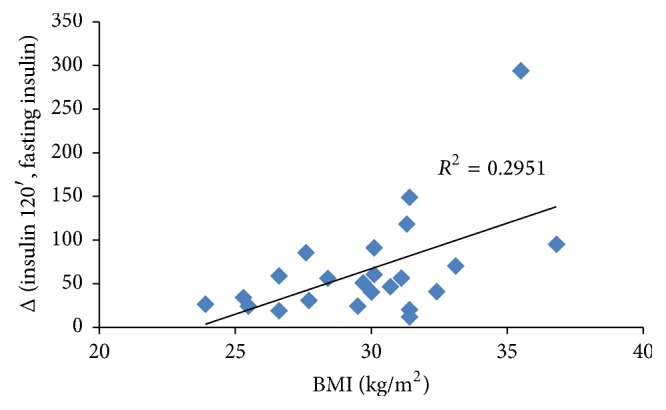
Correlation between BMI and insulin increase from fasting to 120 minutes after OGTT without inositol in the whole sample. *p* = 0.0336.

**Figure 2:**
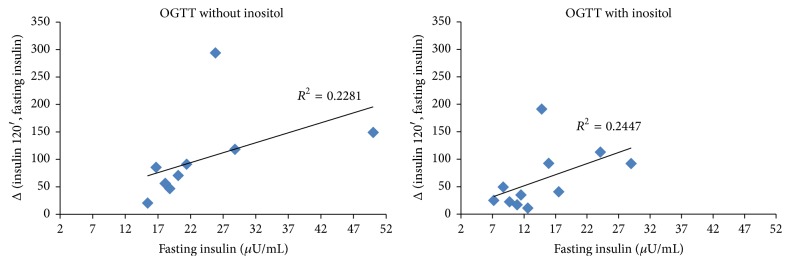
Correlation between basal insulin level and insulin increase from fasting to 120 minutes after OGTT without or with inositol in subjects with a basal insulin level ≥15 *μ*U/mL. (a) *p* = 0.0009. (b) *p* = 0.0767.

**Figure 3:**
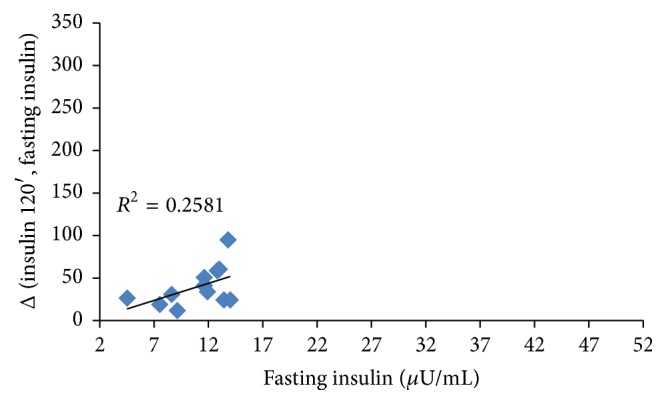
Correlation between basal insulin level and insulin increase from fasting to 120 minutes after OGTT without inositol in subjects with a basal insulin level <15 *μ*U/mL. *p* = 0.2198.

To assess the differences between variables over time we performed the Wilcoxon signed rank sum test assuming *p* ≤ 0.05 as significant level. The Spearman Correlation Coefficient was employed to identify a correlation between BMI and insulin increase after OGTT and between fasting insulin and insulin level after glucose load (Figures 1, 2, and 3). Correlation is used to assess the strength and direction of the linear relationship between two variables. It measures the association, not the casual relationship [14].

## 4. Results and Discussion

### 4.1. Results

Figure 1 shows that the baseline correlation between BMI and insulin increase in the whole sample was statistically significant (*R* ^2^ = 0.2951; *p* = 0.0336), confirming that higher BMI is associated with a greater insulin increase.

Therefore, we performed an OGTT preceded by inositol administration in a subgroup of 11 children only, where the high fasting insulin level (≥15 *μ*U/mL) suggested increasing insulin resistance.

Figure 2 shows the correlation between basal insulin value and its increase after the OGTT performed without inositol and with inositol in the patients with basal insulin ≥15 *μ*U/mL. While correlation was strong and statistically significant in the first case (*R* ^2^ = 0.2281; *p* = 0.0009), it lost significance when the OGTT was preceded by inositol administration (*R* ^2^ = 0.2447; *p* = 0.0767). In this case, basal insulin level and insulin increase got close to those observed in the children who had a basal insulin level ≤15 *μ*U/mL (Figure 3), suggesting that inositol is more effective in lowering insulin increase in children with higher basal insulin level, though this assumption is still uncertain since only a few samples have been tested.

Table 2 reports baseline values and variations of blood glucose and insulin after 120 minutes in the two groups of children, respectively, with insulin level ≥ or ≤15 *μ*U/mL. Both groups recorded a significant increase of glucose and insulin after 120 minutes: in particular, in the first group the mean glucose increase after the OGTT was 29.4 ± 17.9 mg/dL (*p* = 0.001) and mean insulin increase was 93.5 ± 75.9 *μ*U/mL (*p* = 0.001); in the second one mean glucose increase after the OGTT was 12.3 ± 15.0 (*p* = 0.0029) and mean insulin increase was 39.8 ± 23.3 (*p* = 0.0005).

Table 3 shows the comparison between data without inositol and the OGTT performed after inositol administration in the patients with baseline insulin ≥15 *μ*U/mL: fasting glucose was not significantly different from the previous test (88.5 ± 3.4 versus 86.9 ± 3.9 mg/dL) and there was a small nonsignificant reduction of glucose after 120 minutes (117.9 ± 19.2 versus 106.2 ± 18.0 mg/dL), while a reduction of fasting insulin (22.6 ± 10.0 versus 14.7 ± 6.7 *μ*U/mL; *p* = 0.001) was noted, and a decrease of insulin after 120 minutes (116.1 ± 81.4 versus 77.3 ± 58.4 *μ*U/mL; *p* = 0.0176). In addition, in these 11 patients mean BMI after six months was not significantly different from the baseline (31.0 ± 2.1 versus 31.0 ± 2.6 kg/m^2^). In fact, they resulted to have had a BMI variation ≤2.5 kg/m^2^. In particular, some children have obtained a BMI reduction (*n* = 4), stabilization (*n* = 3), or an increase (*n* = 4), in a similar distribution. Thus BMI variation did not influence insulin variations and the results can be attributed to inositol only.

### 4.2. Discussion

The effectiveness of inositol in lowering plasma glucose concentration and postprandial blood glucose levels has been reported in several cases of mellitus diabetes in rats, monkeys, and humans. These studies showed that this effect was related to insulin-sensitizing activity [15–18]. D-Pinitol exerted an acute and chronic insulin-like antihyperglycaemic effect on glucose transport in STZ-diabetic mice, but acute administration of D-pinitol did not significantly alter plasma glucose or insulin concentration over 6 hours in severely insulin-resistant ob/ob mice [19]. When administered to insulin-resistant and diabetic monkeys, D-chiro inositol accelerated glucose disposal and activated glycogen synthase in muscle biopsies beyond that of maximal insulin stimulation [20]. Pinitol was administered to humans with type II diabetes for 13 weeks and it significantly decreased mean fasting plasma glucose and insulin and improved lipid profile, while four weeks of pinitol treatment did not alter insulin-mediated glucose disposal in individuals with obesity and mild type II diabetes [21, 22].

It seems that body weight is not significantly affected by inositol treatment [19, 21] while inositol has been reported to improve insulin sensitivity and ovulatory function in young women affected by polycystic ovary syndrome. When the effect of inositol has been investigated in postmenopausal women with metabolic syndrome and in pregnant women with gestational diabetes, it resulted in improvements of fasting serum insulin and blood glucose levels [23–25]. In particular, in pregnant women with a family history of type 2 diabetes a supplement of myoinositol throughout the pregnancy also reduced the 1-hour glycemia at the OGTT stage and reduced the incidence of gestational diabetes [26].

However, those studies only include women with a specific hormonal status (gestation, menopause, or PCOS) which does not allow us to confirm a similar effect of inositol supplementation in other contexts; therefore the true mechanism of action is still unclear.

To our knowledge this is the first study to test the effect of inositol in children, even though our results are only preliminary.

The main aim of this process was to determine whether an acute effect of inositol on insulin sensitivity in children exists, when administered before a glucose tolerance test.

Our results indicate that insulin increase after glucose intake may be attenuated when inositol is administered before the OGTT. In particular, it seems that inositol reduces insulin increase mainly in children with high basal insulin level. So patients with high fasting insulin might be the ones to benefit more from the administration of inositol.

In our study the observed correlations are not high, but this may be due to the low number of units in the sample analyzed. They seem however indicative of an association between the observed insulin increase and the level of basal insulin that should be analyzed in detail with ad hoc larger studies.

## 5. Conclusions 

Myoinositol and D-chiro inositol have been shown to reduce insulin increase after glucose intake in obese children. Further investigations and larger studies are required to define the physiological and potential therapeutic effects of their administration, but they could be effective oral nonpharmacological agents in the prevention of type II diabetes in children.

## Figures and Tables

**Table 1:** Characteristics of enrolled subjects at baseline.

Variable	*N*	Mean ± SD	Median [25th–75th]
Age (years)	23	11.5 ± 2.3	12.0 [10.0–13.0]
Weight (kg)	23	70.4 ± 14.9	75.5 [56.5–82.7]
Height (cm)	23	153.0 ± 13.3	155.0 [143.0–163.0]
BMI (kg/m^2^)	23	29.8 ± 3.1	30.1 [27.6–31.4]
Fasting glucose (mg/dL)	23	86.7 ± 4.5	87.0 [84.0–89.0]
Fasting insulin (*μ*U/mL)	23	16.6 ± 9.2	14.0 [11.6–18.8]

**Table 2:** Baseline values and changes by baseline insulin level.

	Basal insulin level ≥15 *μ*U/mL				Basal insulin level ≤15 *μ*U/mL			
Variable	*N*	Mean ± SD	Median [25th–75th]	*p* ^*∗*^	*N*	Mean ± SD	Median [25th–75th]	*p* ^*∗*^
Fasting glucose (mg/dL)	11	88.5 ± 3.4	87.0 [86.0–90.0]		12	85.0 ± 4.9	85.0 [82.0–88.0]	
Blood glucose 120′ (mg/dL)	11	117.9 ± 19.2	116.0 [98.0–136.0]		12	97.3 ± 16.2	94.0 [87.0–99.5]	
Δ (blood glucose 120′, fasting glucose)	11	29.4 ± 17.9	27.0 [14.0–46.0]	0.001	12	12.3 ± 15.0	6.5 [3.0–17.5]	0.003
Fasting insulin (*μ*U/mL)	11	22.6 ± 10.0	18.8 [16.7–25.8]		12	11.0 ± 2.9	11.8 [8.9–13.2]	
Insulin 120′ (*μ*U/mL)	11	116.1 ± 81.4	90.6 [65.5–147.0]		12	50.7 ± 24.9	42.7 [34.3–67.1]	
Δ (insulin 120′, fasting insulin)	11	93.5 ± 75.9	70.5 [46.7–118.2]	0.001	12	39.8 ± 23.3	32.5 [24.2–54.9]	0.000

^*∗*^Wilcoxon signed rank sum test.

**Table 3:** Values at baseline (OGTT without inositol) and after 6 months (OGTT with inositol) of the subjects with a basal insulin level ≥15**** *μ*U/mL.

	OGTT without inositol		OGTT with inositol		
Variable	Mean ± SD	Median [25th–75th]	Mean ± SD	Median [25th–27th]	*p* ^*∗*^
BMI (kg/m^2^)	31.0 ± 2.1	31.1 [30.0–31.4]	31.0 ± 2.6	31.4 [29.8–32.5]	0.867
Weight (kg)	72.1 ± 15.7	75.9 [56.3–83.4]	74.4 ± 16.0	78.9 [60.0–84.8]	0.201
Fasting glucose (mg/dL)	88.5 ± 3.4	87.0 [86.0–90.0]	86.9 ± 3.9	87.0 [83.0–90.0]	0.177
Blood glucose 120′** **(mg/dL)	117.9 ± 19.2	116.0 [98.0–136.0]	106.2 ± 18.0	106.0 [93.0–113.0]	0.101
Fasting insulin (*μ*U/mL)	22.6 ± 10.0	18.8 [16.7–25.8]	14.7 ± 6.7	12.6 [9.7–17.5]	0.001
Insulin 120′** **(*μ*U/mL)	116.1 ± 81.4	90.6 [65.5–147.0]	77.3 ± 58.4	57.9 [32.0–121.0]	0.017

^*∗*^Wilcoxon signed rank sum test.
